# Supplementary material for: Variation in spend on young mental health across Clinical Commissioning Groups in England: a cross-sectional observational study
Source: BMJ Open. 2019 Oct 7;9(10):e030011. doi: 10.1136/bmjopen-2019-030011 (PMC6797274; doi:10.1136/bmjopen-2019-030011)
Supplement: Supplementary data [file bmjopen-2019-030011supp001.pdf]

Appendix

Table 1 - Overview of data

Variable	Category	Year	Source	Note
Spend on CYP MH	Dependent variable	2016/17	Mental Health Five Year Forward View Dashboard	
Estimated prevalence (aged 5-16) of:<br>• any mental health condition<br>• conduct disorder<br>• emotional disorders<br>• hyperkinetic disorders	CYP MH need	2015 (2004 prevalence estimate applied to 2015 population)	PHE Fingertips	
Children in need	CYP MH need	2016/17	Gov.uk, Characteristics of children in need: 2016 to 2017	Estimated from local authority data*
Hospital admissions as a result of self-harm among those aged 0-24	CYP MH need	2016/17	PHE Fingertips	
Hospital admissions for mental health under 18 (rate per population aged 0-18)	CYP MH need	2015/16, 2016/17	PHE Fingertips	
Child Development at age 5	CYP MH need	2013/14	Local Health	
GCSE Achievement 5 A-C	CYP MH need	2013/14	Local Health	
Obese Children (Reception Year)	CYP MH need	2013/14-2015/16	Local Health	
Children with excess weight (Reception Year)	CYP MH need	2013/14-2015/17	Local Health	
Children with excess weight (Year 6)	CYP MH need	2013/14-2015/18	Local Health	
Obese Children Year 6	CYP MH need	2013/14-2015/16	Local Health	
Regular smoker (modelled prevalence)	CYP MH need	2009-2012	Local Health	
Looked after children where there is a cause for concern	CYP MH need	2016/17	PHE Fingertips	Estimated from local authority data*
Children leaving care	CYP MH need	2016/17	PHE Fingertips	Estimated from local authority data*
Children in care	CYP MH need	2016/17	PHE Fingertips	Estimated from local authority data*
Children entering youth justice system	CYP MH need	2016	PHE Fingertips	Estimated from local authority data*
Children under 16 in poverty (%)	CYP MH need	2015	PHE Fingertips	Estimated from local authority data*
Family homelessness (%)	CYP MH need	2016/17	PHE Fingertips	Estimated from local authority data*

Not in education, employment or training (%)	CYP MH need	2015	PHE Fingertips	Estimated from local authority data*
Special educational needs (SEN) (rate per population aged 0-18)	CYP MH need	2016	Gov.uk, Special educational needs (SEN)	Estimated from local authority data*
Adult mental health spend (excluding CYP MH, but including learning disabilities)	Adult MH need	2016/17	Mental Health Five Year Forward View Dashboard	
Quality and outcomes framework (QOF) prevalence estimates (%):<br>• Depression (ages 18+)<br>• Severe Mental Illness	Adult MH need	2015/16	NHS Digital	
Mental health total primary care expenditure (£000)	Adult MH need	2015/16	NHS RightCare 'Where to look' packs	
Quality and outcomes framework (QOF) prevalence estimates (%):<br>• Asthma<br>• Cancer<br>• Chronic Kidney Disease (CKD) (ages 18+)<br>• Chronic Obstructive Pulmonary Disease (COPD)<br>• Coronary heart disease (CHD)<br>• Diabetes mellitus (ages 17+)<br>• Dementia<br>• Epilepsy<br>• Learning Disabilities<br>• Heart Failure<br>• Hypertension<br>• Obesity (ages 16+)<br>• Osteoporosis<br>• Palliative care<br>• Peripheral arterial disease<br>• Rheumatoid Arthritis<br>• Stroke and transient ischaemic attack (STIA)<br>• Atrial fibrillation	Physical health need	2015/16	NHS Digital	
1) Spend on physical health conditions (2015/16) – age and sex standardised:<br>o Asthma (non-elective)<br>o Cancer:<br>i. Bowel cancer (electives, non-elective)<br>ii. Lung cancer (electives, non-elective)<br>iii. Breast cancer (elective, primary care)<br>o Endocrine (electives, non-elective, primary care)<br>o Gastrointestinal (electives, non-elective, primary care)<br>o Genitourinary (electives, non-elective, primary care)<br>o Maternity (primary care)<br>o Musculoskeletal (electives, non-elective, primary care)<br>o Neurological (electives, non-elective, primary care)<br>o Respiratory (electives, non-elective, primary care)<br>o Trauma and injuries (electives, non-elective, primary care)	Physical health need	2015/16	NHS RightCare 'Where to look' packs	
Elective hospital admissions for all causes	Physical health need	2011/12-2015/16	Local Health	
Emergency hospital admissions for all causes	Physical health need	2011/12-2015/16	Local Health	
Indices of multiple deprivation (IMD)	Confounder	2015	Local Health	

Total CCG core budget allocation	Confounder	2016/17, 2017/18	NHS England, Allocations	
BME	Confounder	2011	Local Health	
Population whose ethnicity is not White British	Confounder	2011	Local Health	
Long Term Unemployment	Confounder	2016/17	Local Health	
Unemployment	Confounder	2016/17	Local Health	
% Population aged 65+	Confounder	2016/17	Local Health	
% Population aged 85+	Confounder	2016/17	Local Health	
Fuel Poverty	Confounder	2016/17	Local Health	
General Health bad or very bad	Confounder	2016/17	Local Health	
Hospital stays for alcohol related harm	Confounder	2016/17	Local Health	
Overcrowding	Confounder	2016/17	Local Health	
MFF Index	Confounder	2016/17	NHS England, Allocations	

*** Mapping Local Authorities to CCGs**

Local Government Association:
https://www.local.gov.uk/sites/default/files/documents/mapping-ccgs-hwbs-and-hwb-507.xls

Table 2 - Descriptive statistics of explanatory factors included in the main regression model

	count	mean	sd	min	max
CYP MH spend per person aged 0-18	209	46.39	18.51	2.25	127.42
Children entering youth justice per 1,000 aged 0-18	204	3.23	1.20	0.98	7.40
Children in need, Primary need: family stress, rate per 100 aged 0-18	204	1.02	0.58	0.11	2.70
Obese Children (Reception Year)	204	9.25	1.58	5.30	13.80
Children with excess weight (Reception Year)	204	22.10	2.35	14.60	27.20
Children in care per 1,000 aged 0-18	204	6.13	2.23	2.15	16.38
Spend on MH per person over 18 (excl. CYP, £10)	209	22.45	5.34	12.66	44.41
All physical health spend per person (£100)	209	5.80	1.01	3.44	9.26
Black and minority ethnic (%)	204	14.03	15.53	1.20	72.20
Overcrowding	204	8.68	7.01	2.70	34.90
Long term unemployment (%)	204	3.57	2.54	0.50	14.40
