# Supplementary material for: Variation in spend on young mental health across Clinical Commissioning Groups in England: a cross-sectional observational study
Source: BMJ Open. 2019 Oct 7;9(10):e030011. doi: 10.1136/bmjopen-2019-030011 (PMC6797274; doi:10.1136/bmjopen-2019-030011)
Supplement: Author's manuscript [file bmjopen-2019-030011.draft_revisions.pdf]

BMJ Open is committed to open peer review. As part of this commitment we make the peer review history of every article we publish publicly available.

When an article is published we post the peer reviewers' comments and the authors' responses online. We also post the versions of the paper that were used during peer review. These are the versions that the peer review comments apply to.

The versions of the paper that follow are the versions that were submitted during the peer review process. They are not the versions of record or the final published versions. They should not be cited or distributed as the published version of this manuscript.

BMJ Open is an open access journal and the full, final, typeset and author-corrected version of record of the manuscript is available on our site with no access controls, subscription charges or pay-per-view fees (http://bmjopen.bmj.com).

If you have any questions on BMJ Open's open peer review process please email info.bmjopen@bmj.com

BMJ Open

Budget allocation at the commissioning level: does child mental health lose out?

Journal:	*BMJ Open*
Manuscript ID	bmjopen-2019-030011
Article Type:	Research
Date Submitted by the Author:	22-Feb-2019
Complete List of Authors:	Rocks, Stephen; University of Oxford, Health Economics Research Centre<br>Fazel, Mina; University of Oxford, Warneford Hospital,, Psychiatry; University of Oxford<br>Tsiachristas, Apostolos; University of Oxford, Health Economics Research Centre
Keywords:	Health economics < HEALTH SERVICES ADMINISTRATION & MANAGEMENT, Organisation of health services < HEALTH SERVICES ADMINISTRATION & MANAGEMENT, MENTAL HEALTH, Child & adolescent psychiatry < PSYCHIATRY

SCHOLARONE™
Manuscripts

Budget allocation at the commissioning level: does child mental health lose out?

Authors
Stephen Rocks[1 x], Mina Fazel[2], Apostolos Tsiachristas[1]

[x] Corresponding author
stephen.rocks@ndph.ox.ac.uk
+44 (0)1865 289470

[1] Health Economics Research Centre, Nuffield Department of Population Health, University of Oxford, Richard Doll Building, Old Road Campus, Oxford OX3 7LF
[2] Department of Psychiatry, University of Oxford, Warneford Hospital, Warneford Ln, Oxford OX3 7JX

Word count – 3,495 (excluding title page, abstract, references, figures, tables, acknowledgements, contributions and references)

ABSTRACT

Objectives
To investigate whether the rate of spend on child and adolescent mental health is influenced by demand for other competing services in local commissioning decisions.

Design
Analysis of spend data by CCG, including other publicly available data to control for variation in need.

Setting
Local commissioning decisions in the NHS.

Participants
Commissioning of health services across 209 Clinical Commissioning Groups (CCGs).

Main outcome measures
Association between the rate of child and adolescent mental health spend and demand for CAMHS, adult mental health services and physical health services after adjusting for confounding factors.

Results
Spending £100 more on physical health per person is associated with an 11% (95%CI: 17%-4%) decrease in spend on child and adolescent mental health, while a £10 increase in adult mental health spend is associated with a 2% increase (95%CI: 0%-3%) in spend on child and adolescent mental health.

For peer review only - http://bmjopen.bmj.com/site/about/guidelines.xhtml

For peer review only

Conclusions

This study empirically supports current concerns that child and adolescent mental health is poorly prioritised when compared with physical health in local budget decisions in England. Ring-fencing funding, raising awareness among commissioners and improving the evidence base are steps that could contribute to more equitable funding in this area of need.

Strengths and limitations of this study

- This is the first study looking broadly at the factors associated with the commissioning of child and adolescent mental health services.
- A major strength is the incorporation of different area level data.
- However, there is limited data available for spend at a commissioning level.
- There are also concerns over the consistency of published figures for spend on child and young person mental health.
- The analytic approach and incorporation of sensitivity analyses strengthen this study.

For peer review only

For peer review only - http://bmjopen.bmj.com/site/about/guidelines.xhtml

Introduction

Mental health problems imposes a significant burden on individuals, their families, and society.[1] In the UK, it is estimated that the costs associated with mental illness totals 4.5 percent of gross domestic product (GDP), driven mainly by productivity loss and opportunity cost of informal care.[2] Childhood and adolescence is crucial to any attempt to alleviate this burden because the majority of lifelong mental illness occurs by the age of 24,[3] exerting short and long-term costs that impact significantly across the life course.[4] However, although effective interventions exist,[5] most people do not receive treatment during their childhood and adolescence.[3,6] It is estimated that less than a quarter of young people with a mental health problem receive any help from specialist services.[3] This treatment gap reflects the disparity between the needs and resources committed to the mental health of young people.

In England, the decision on how to fund most child and adolescent mental health services (CAMHS) is taken by NHS Clinical Commissioning Groups (CCGs). However, the proportion of spend they allocate is not predetermined. CCGs are assigned an overall budget based on need to commission a range of health services for their local population, but as NHS England states, 'It is for CCGs to decide their priorities for spending'.[7] Recent evidence suggests that budget allocation to CAMHS does not reflect levels of psychopathology. For instance, between 2012/13 and 2014/15, there was a sharp rise in referrals to CAMHS,[8] alongside a growing number of young people presenting at A&E with self-harm,[9] but budgets for CAMHS were reduced in real terms, resulting in reports of rises in thresholds for care, longer waiting times, and considerable variations in local service provision.[10] Although the Government recently earmarked additional national funding for CAMHS, there is concern that pressure across the health system means funds may be diverted to other areas.[10] Indeed CAMHS has been described as the 'Cinderella of the Cinderella service',[8] in that despite repeated promises of additional funding it has remained under-resourced relative to levels of need.

This raises the question as to whether expenditure might be biased towards other conditions or groups, in particular towards the needs of older people and/or physical health conditions. One way to assess this is to examine the spending decisions taken by CCGs in England. Analysis by the Royal College of Psychiatrists discovered a stark variation in spend on CAMHS, contributing to a "postcode lottery" in access to services.[11] This may be justified if the variation in spend reflected differences in need, but research suggests it is only weakly associated with deprivation – a proxy for need - with 'stark unexplained variation'.[11] Instead, it could be that spend on CAMHS reflects demand for other competing services, either adult mental health or in particular general or acute health services. With rising demand across the NHS for both mental and physical health services, it is important to understand whether CAMHS is consistently overlooked in funding decisions, possibly because short-term pressures take priority over early interventions for adolescents, for whom interventions can prevent long-term negative trajectories.[5]

Methods

Data sources

Data was accessed from publicly available governmental sources. Data on spend for child and young person mental health (CYP MH) and adult mental health by CCG (N=209) was available from the Five

For peer review only - http://bmjopen.bmj.com/site/about/guidelines.xhtml

Year Forward View Mental Health Dashboard (FYFVMHD).[12,i] Annual spend figures were available for the financial years 2016/17 and 2017/18. CCG population data was sourced from the Office of National Statistics (ONS).

Other data on CCG areas were identified from a range of publicly available UK data sets, including Public Health Fingertips, the NHS Outcomes Tool, NHS RightCare packs, and Local Health. As far as possible data was used for the same year as CYP MH spend (2016/17). Where this was not available, the nearest possible date was used; 2016 was chosen where data was available for calendar years. When data were reported for local authorities rather than CCGs, approximate values for CCGs were calculated using a 'lookup' file from the Local Government Association, which provides a link between local authority and CCG data. A full list of accessed data with time periods and sources is provided in the supplementary file (table 1).

Spend on child and young person mental health

CCG reporting on CYP MH spend was introduced as part of the FYFVMHD. This spend figure does not include in-patient services which are commissioned directly by NHS England, but does include the majority of CAMHS spend on tiers 2 (early help and targeted services) and 3 (specialised CAMHS services).[13] Spend on specialist eating disorder services is reported separately, and spend on services directly commissioned by other sources, such as some children's psychological medicine or liaison services commissioned by acute healthcare trusts, are not included.

We used 2016/17 spend in our analysis given the availability of data in other categories of our analysis. Spend was analysed in absolute terms and as a rate per young person aged 0 to 18, using CCG Mid-Year Population Estimates from the Office of National Statistics. [11]

Potential explanatory factors of CAMHS spend variation

We selected a comprehensive set of indicators using a mix of evidence and consultation with health economic and mental health experts.

Category A: Demand for CAMHS

We included 2015 local prevalence estimates for mental health conditions as an indicator of demand for CAMHS. In-patient admissions to psychiatric units among those aged under-18 and rates of self-harm were also included as proxies of need. We also included other potential explanatory factors, such as being a regular smoker at age 15, which is positively associated with child and adolescent mental health conditions;[14] the rate of children "in need"[15], the rate of children in care; as well as a range of broader measures of vulnerability amongst young people, as detailed in Table 1.

Category B: Demand for adult mental health services

For adult mental health, we included 2016/17 mental health spend (excluding spend on children and young people and including primary care spend on mental health) from the FYFVMHD, 2015/16 mental health primary care expenditure (£000) from NHS RightCare 'Where to Look' packs and, finally, QOF prevalence estimates for Depression and Severe Mental Illness from 2015/16.

Category C: Demand for physical health services

[i] NHS England reports on 'child and young person' mental health (CYP MH) spend. CCGs may spend small amounts on other services for CYP MH, but in this paper we take this as a proxy for local spend child and adolescent mental health services (CAMHS).

For peer review only - http://bmjopen.bmj.com/site/about/guidelines.xhtml

The need for physical health spend was proxied by a combination of spend, hospital admissions and prevalence rates. We included a range of physical health spend figures from 2015/16 as reported in NHS RightCare 'Where to Look' packs. In the packs, spend was reported based on major International Classification of Disease (ICD-10) codes, further broken down into elective, non-elective and primary care spend and expressed as standardised rates. To calculate the total physical health spend rate, we summed the numerators (absolute spend) across all physical health categories and expressed this as a rate using the respective CCG population. For the more detailed model we summed the numerators for elective, non-elective and primary care spend for each condition and expressed this as a rate using the CCG population. We also included rates of elective and emergency hospital admissions. In addition, QOF prevalence estimates from 2015/16 were used as alternative indicators of demand for physical health care. We chose prevalence estimates of this year because they were available at the point of the commissioning decisions in 2016/17 and were expected to be associated with the variable of interest.

Category D: General factors

More general factors associated with health needs and spend on CYP MH were also included in the analysis. This included the level of deprivation, as measured by the index of multiple deprivation (IMD), unemployment, and long-term unemployment. Research also suggests ethnic minority status may be linked to unmet need - the Children's Commissioner found children of Asian background were under-represented in CAMHS -[16] therefore the proportion of the population from black and minority ethnic (BME) groups was considered, as well as the population without White-British ethnicity. The core allocation per person for each CCG was included to control for differences in budget. We also included the difference between what NHS England's allocation formula suggests an area should get (target allocation) and the actual amount allocated in a given year – the 'distance from target allocation'. The two values diverge because of rules limiting the pace of change i.e. the amount by which allocations can change year-on-year.

Table 1 – Summary of potential explanatory factors of CAMHS spend variation

**Category A: Demand for CAMHS**
• Prevalence estimates (% aged 5-16), of: any mental health condition, conduct disorder, emotional disorders, hyperkinetic disorders<br>• Child development at age 5 (%)<br>• GCSE Achievement (%) – attaining 5 A-Cs including English and Maths<br>• Child obesity (%), at: reception year, year 6<br>• Children in need (rate per population aged 0-18): Episodes per year, Children per year, Rate where mental health is a factor, Rate where domestic violence is a factor, Rate where cause is abuse or neglect, Rate where cause is family stress<br>• Special educational needs (SEN) (rate per population aged 0-18)<br>• Children overweight (%), at: reception year, year 6<br>• Hospital admissions for mental health under 18 (rate per person aged 0-18)<br>• Hospital admissions as a result of self-harm, population aged 10-24 (directly standardised rate)<br>• Children in care (rate per population aged 0-18): Children in care, Children leaving care<br>• Looked-after-children where there is a cause for concern (rate per person aged 0-18)<br>• Children under 16 in poverty (%)<br>• Family homelessness (%)<br>• Not in education, employment or training (%)

For peer review only - http://bmjopen.bmj.com/site/about/guidelines.xhtml

• Rate of first time entrants to the youth justice system
**Category B: Demand for adult mental health services**
• Quality and outcomes framework (QOF) prevalence estimates (%): Depression, Severe Mental Illness<br>• Adult mental health spend (excluding CYP MH, but including learning disabilities)<br>• Mental health total primary care expenditure (£000)
**Category C: Demand for physical health services**
• All physical health spend per person (2015/16)<br>• Hospital admissions: Elective and emergency hospital admissions for all causes<br>• Physical health spend per person (2015/16), by condition: Asthma, Cancer (Bowel, Lung, Breast, Other), Circulatory conditions (Cerebrovascular disease, Coronary heart disease, Other), Endocrine (Diabetes, Other), Gastrointestinal (Liver, Lower GI, Upper GI, Other), Genitourinary (Renal, Other), Maternity, Musculoskeletal, Neurological, Osteoarthritis, Osteoporosis, Respiratory (Asthma, Obstructive, Other) Admissions relating to fractures where a fall occurred, Trauma and injuries<br>• Quality and outcomes framework (QOF) prevalence estimates (%) for: Asthma, Cancer, Chronic Kidney Disease (CKD), Chronic Obstructive Pulmonary Disease (COPD), Diabetes, Dementia, Epilepsy, Learning Disabilities, Heart Failure, Hypertension, Obesity, Osteoporosis, Rheumatoid Arthritis, Palliative care, Peripheral arterial disease, Coronary heart disease (CHD), Stroke and transient ischaemic attack (STIA), Atrial fibrillation
**Category D: General factors**
• Index of multiple deprivation (IMD)<br>• Unemployment: Unemployment (%), Long term unemployment (%)<br>• Ethnicity: BME (%), Population whose ethnicity is not 'White UK' (%)<br>• Total CCG core allocation per capita<br>• Distance from target allocation (DfT)

For peer review only

Statistical analysis

To explain variation in spend on CYP MH across CCGs, we reviewed the proportion of CCG budgets spent on CYP MH before inspecting the association between the proportion spent on CYP MH and the total CCG budget, as well as the association between CYP MH spend per CCG and the population of the CCG aged 0-18.

To investigate statistical associations between CYP MH spend per person aged 0-18 and the explanatory factors identified we followed a two-step analytical process. First, we selected factors from each category listed in Table 1 that were associated with the dependent variable (i.e. spend on CYP MH per person aged 0-18). To do this, we included all factors in each category in a generalised linear model (GLM) and used likelihood ratio tests to select backwards those factors that gave the best goodness-of-fit of the model to the data. The order in which the factors were tested by likelihood ratio tests was determined by the p-value from the regression (from largest to smallest p-value). Second, all selected factors from each category were included in a GLM and the regression coefficients were presented in the exponentiated form to give the ratio of means (i.e. the proportional adjustment in the dependent variable "child and young person mental health spend per person aged 0-18" expected from a 1-unit increase in the covariate). All GLMs were specified with Gamma distribution and log link function because this combination provided the best goodness-of-fit based on a Modified Park Test (using the user-command "GLMdiag" in STATA) and the AIC and BIC

For peer review only - http://bmjopen.bmj.com/site/about/guidelines.xhtml

information criteria. We ran the analysis in the main model featuring the rate of all physical health spend. To understand the relationship in detail, we also ran a second, detailed physical care model, which included spend broken down by category.

Sensitivity analyses

To address the uncertainty in the results, we conducted three sensitivity analyses. First, we removed CCGs with extreme low and high spend on CAMHS from the analysis to address concerns that CAMHS spend figures are inappropriately recorded for some CCGs.[17] The extreme CAMHS spend values (or outliers) were identified using studentized deviance residuals. Second, we added spend on eating disorders in the dependent variable (i.e. spend on CYP MH per person aged 0-18) because some CCGs have been unable to split out these costs due to block contracting and it was unclear if, for some CCGs, these costs were excluded entirely from the dependent variable.[10] Third, QOF prevalence estimates were used as indicators of physical health need instead of spend on physical care because physical health spend data was out of date by a year compared with the CAMHS spend data.

Patient and public involvement

This research was done without patient involvement. Patients were not invited to comment on the study design and were not consulted to develop patient relevant outcomes or interpret the results. Patients were not invited to contribute to the writing or editing of this document for readability or accuracy.

Results

Spend on CYP MH (excluding spend on specialist eating disorder services) accounted on average for 0.8% of CCGs' budget in 2016/17. As shown in Figure 1, it appears that the proportion of CCG budget spent on CYP MH has a weak positive association with the size of the CCG budget and a strong positive association with the size of the CYP population.

Figure 1 – Scatter plots: Spend on child and young person mental health services

The average spend on CYP MH per person aged 0-18 in 2016/17 across 209 CCGs was £46 (SD=£18, min=£2, max=£127) with considerable variation across CCGs in England (Figure 2).

Figure 2 – Spend on child and young person mental health services, by CCG

The results from the main regression analysis are presented in Table 2 and show that an additional percentage point in the proportion of children in care is associated with 3% higher CYP MH spend per person aged 0-18 (ratio of means: 1.03; 95%CI: 1.00, 1.07) and spending £10 more on adult mental health is associated with 2% more spend on CYP MH spend per person aged 0-18 (ratio of means: 1.02; 95%CI: 1.00, 1.03). In contrast, spending £100 more on physical health services was associated with 11% lower spend in CYP MH per person aged 0-18 (ratio of means: 0.89; 95%CI: 0.83, 0.96). The proportion of the population of black and minority ethnicity also appeared to be negatively associated with the dependent variable but the magnitude was small and of borderline statistical significance (ratio of means: 0.99; 95%CI: 0.99, 1.00). Overall, the main regression model explained 29% of the variation in CYP MH spend per person. The summary statistics for the

For peer review only - http://bmjopen.bmj.com/site/about/guidelines.xhtml

explanatory factors included in the main regression model are presented in the supplementary file (table 2).

With regards to the first sensitivity analysis, 7 CCGs were excluded because they spent less than £11 per person aged 0-18 on CYP MH and 2 CCGs were excluded because they spent above £117 per head. The results of this sensitivity analysis as well as the sensitivity analysis where spend on eating disorders was added in the dependent variable were very similar to the results of the main analysis. The only noticeable difference was that the size of the negative association of spend on physical health with the dependent variable was reduced from 11% in the main analysis to 10% (ratio of means: 0.90; 95%CI: 0.84, 0.97) and 8% (ratio of means: 0.92; 95%CI: 0.86, 0.98) respectively.

For peer review only

For peer review only - http://bmjopen.bmj.com/site/about/guidelines.xhtml

Table 2 – Association of CYP MH spend per person aged 0-18 with explanatory factors (main model)

	Main analysis<br>Ratio of Means (SE) [95% CI]	SA 1 – excluding outliers<br>Ratio of Means (SE) [95% CI]	SA 2 – including eating disorders<br>Ratio of Means (SE) [95% CI]
Children entering youth justice per 1000 aged 0-18	1.04 (0.03) [0.99,1.10]	1.02 (0.02) [0.98,1.07]	1.04 (0.03) [0.99,1.09]
Children in need (family stress), rate per 100 aged 0-18	1.06 (0.05) [0.97,1.16]	1.05 (0.04) [0.97,1.13]	1.07 (0.05) [0.98,1.16]
Obese Children (Reception Year)	0.95 (0.06) [0.84,1.06]	0.96 (0.05) [0.87,1.05]	0.95 (0.05) [0.85,1.06]
Children with excess weight (Reception Year)	1.05 (0.04) [0.98,1.12]	1.05 (0.03) [0.99,1.11]	1.04 (0.03) [0.98,1.11]
Children in care per 1,000 aged 0-18	1.03* (0.02) [1.00,1.07]	1.03* (0.01) [1.00,1.05]	1.03* (0.02) [1.00,1.07]
Spend on MH per person over 18 (excl. CYP, £10)	1.02* (0.01) [1.00,1.03]	1.02** (0.01) [1.01,1.03]	1.02* (0.01) [1.00,1.03]
All physical health spend per person (£100)	0.89** (0.03) [0.83,0.96]	0.92** (0.03) [0.86,0.98]	0.90** (0.03) [0.84,0.97]
% Black and minority ethnic	0.99* (0.00) [0.99,1.00]	0.99* (0.00) [0.99,1.00]	0.99* (0.00) [0.99,1.00]
Long term unemployment	1.02 (0.01) [0.99,1.05]	1.02 (0.01) [0.99,1.04]	1.02 (0.01) [0.99,1.05]
Observations	199	190	199
AIC	1933	1853	1962
BIC	1966	1885	1995
Pseudo R-squared	.29	.30	.28

SA = Sensitivity analysis; * p<0.05, ** p<0.01, *** p<0.001

For peer review only - http://bmjopen.bmj.com/site/about/guidelines.xhtml

The results from the detailed physical care regression model are presented in Table 3 and show that only spend on circulatory conditions (0.98; 95%CI: 0.96, 0.99) was associated with the rate of spend on CYP MH. Specifically, an additional Pound spent on 'other circulatory conditions' per person was associated with a 2% decrease in spend per head on CYP MH. When including QOF prevalence estimates in the detailed model instead of spend on physical health (third sensitivity analysis), the results showed that an additional percentage point in diabetes prevalence was associated with 10% less spend on CYP MH (ratio of means:0.90; 95%CI:0.85, 0.96).

Table 3 – Association of CYP MH Spend per person aged 0-18 with explanatory factors (detailed physical care model)

	**Detailed spend** Ratio of Means (SE) [95% CI]	**SA 3 – QOF prevalence** Ratio of Means (SE) [95% CI]
Children entering youth justice per 1000 aged 0-18	1.03 (0.03) [0.98,1.09]	1.04 (0.03) [0.99,1.10]
Children in need (family stress), rate per 100 aged 0-18	1.08 (0.05) [0.98,1.19]	1.07 (0.05) [0.97,1.17]
Obese Children (Reception Year)	0.95 (0.06) [0.85,1.08]	0.96 (0.06) [0.85,1.08]
Children with excess weight (Reception Year)	1.04 (0.04) [0.97,1.12]	1.05 (0.04) [0.98,1.12]
Children in care per 1,000 aged 0-18	1.03 (0.02) [1.00,1.06]	1.02 (0.02) [0.99,1.06]
Spend on MH per person over 18 (excl. CYP, £10)	1.01 (0.01) [1.00,1.03]	1.01 (0.01) [1.00,1.02]
Spend on circulatory disease per person: coronary heart disease	1.00 (0.01) [0.98,1.03]	
Spend on circulatory disease per person: other	0.98** (0.01) [0.96,0.99]	
Spend on respiratory disease per person: obstructive conditions	1.01 (0.02) [0.98,1.04]	
Spend on cancer per person: bowel cancer	1.02 (0.04) [0.95,1.10]	
Spend on maternity per person	1.18 (0.11) [0.99,1.41]	
% Black and minority ethnic	0.99 (0.00) [0.99,1.00]	0.99 (0.01) [0.98,1.00]
Long term unemployment	1.02 (0.02) [0.99,1.05]	1.02 (0.02) [0.99,1.05]
Cancer prevalence - 18+, QOF		0.93 (0.09) [0.77,1.13]
Diabetes prevalence - 18+, QOF		0.92* (0.03) [0.86,0.99]
COPD prevalence, QOF		0.99 (0.09) [0.83,1.19]
Observations	199	199
AIC	1940	1937
BIC	1986	1976
Pseudo R-squared	.32	.29

SA = Sensitivity analysis; * p<0.05, ** p<0.01, *** p<0.001

Discussion

Our findings indicate that healthcare commissioners in England face a challenge in balancing competing needs and there appears to be a trade-off between physical health and CYP MH when allocating budgets. This trade-off persists when local needs are taken into account, implying something beyond the variation expected under a devolved healthcare commissioning structure. This unintended and undesired situation may be explained by the combination of two possible components of the current commissioning process. The first is that the budgets allocated to CCGs from NHS England do not seem to reflect the real need for physical and mental health services. The second is that CYP MH does not appear to be readily prioritised by commissioners. Several factors may contribute to these possible failures.

The lower quality and visibility of data relating to CYP MH compared to care for adults may influence spending decisions.[18] Historically, there has been a more systematic and thorough recording and measurement of processes relating to acute health conditions such as A&E waiting times and hospital length of stay. However, faced with a relative paucity of data and poor collation of process and outcome data assessing the reach and impact of CAMHS, there may be less pressure on commissioners to prioritise investments in CYP MH.

For peer review only - http://bmjopen.bmj.com/site/about/guidelines.xhtml

Moreover, spending decisions may be biased towards a 'rule of rescue', which predicts that spend will gravitate towards cases of immediate and pressing need and away from what might be considered preventative.[19] Particularly given that spend on CYP MH as used in our study does not include inpatient care, where some of the most severe cases are treated, it may be that CYP MH is perceived as an area where rationing by deterrence or delay is more acceptable than in other areas. Indeed, long waits for CAMHS appear to evidence this.[10]

The general level of awareness or stigma around mental health may also contribute to low prioritisation of CYP MH.[20] The positive association between spend on adult and CYP mental health found in this paper could be explained by commissioners with increased awareness or knowledge of mental health spending more on mental health services for both CYP and adults. Furthermore, the negative association of spend on CYP MH with the proportion of the BME population found in this study may reflect lower levels of awareness of mental health in BME communities and imply that population level awareness of mental health may influence service provision.[21]

Furthermore, we found that spend on CYP MH was negatively associated with spend in circulatory conditions and prevalence of diabetes, which are two of the highest spend disease groups in the UK.[22] Better prevention and early intervention in the community for these disease groups may release resources to help meet the increasing demand for mental health services and in particular CAMHS. Targeting common risk factors for diabetes and for circulatory disease (e.g. obesity and physical inactivity),[23] could potentially reduce in the long run the trade-offs that commissioners may be forced to make between spend in these conditions and CYP MH.

The findings of this paper support several recommendations going forward. One is to improve the quality and availability of data relating to CYP MH. Work is already underway. For instance, recently published estimates of the prevalence of mental health disorders among young people may better capture variation in need.[24] Indeed, the Children's Commissioner has called for new prevalence estimates to be used to better allocate CYP MH funding.[25] However, more data may not be a panacea, especially as research suggests CCGs struggle to make full use of information about population need.[26]

A second recommendation is to intervene to prompt CCGs to better consider CYP MH needs. The most rigid approach would be to ring-fence a given proportion of funding distributed to CCGs for CYP MH.[10] Although short of explicit ring-fencing, CYP MH spend is now being monitored as part of the FYFVMH dashboard; in addition, new targets are being introduced, such as for access rates and waiting times, to help commissioners understand how well services are performing.[12] This approach chimes with long-standing research that finds that commissioning priorities emerge from 'pluralistic bargaining…modified by shifting political judgments'.[27] However, the top-down nature of this approach limits the accountability and flexibility of local commissioners, whilst relying on the identification of appropriate targets.

A broader agenda would be to engage stakeholders, particularly commissioners, in order to build awareness and ensure evidence can meaningfully inform decisions on priority setting. Engaging stakeholders in a process that sets out competing alternatives can at least make explicit the basis for funding allocations, whilst building an understanding of the trade-offs at stake.[28] This deliberative approach should be informed by evidence that is relevant and usable for commissioners.[29] In

For peer review only - http://bmjopen.bmj.com/site/about/guidelines.xhtml

particular, future research should focus on the outcomes arising from different levels of investment in CAMHS and other services that help support young people.

The main limitation of this paper related to the lack of contemporaneous and consistent data. Some of the data were from two years previous, in particular spend on physical health conditions (2015/16), albeit the distribution of spend across different programmes is unlikely to have altered dramatically. Other indicators are not available at CCG level but are estimated from local authority data, specifically this includes a range of factors explaining demand for CYP MH services. Finally, as highlighted above, there are concerns over the consistency and accuracy of the figures published for spend on CYP MH, with challenges in breaking out different facets of expenditure. However, this is expected to have minor impact on the results as indicated by the sensitivity analyses. The major strength of this study is how we have been able to use and combine different area level data from a range of datasets, including efforts to incorporate local authority data to understand factors driving need for CYP MH services. Recent research into variation in Medicare spend in the USA found that area level factors were the most appropriate way to analyse such variation.[30] The sophisticated analytic approach and the incorporation of sensitivity analyses to address uncertainty in the results also strengthen this study.

Conclusions

This study empirically supports current concerns that child and adolescent mental health is poorly prioritised when compared with physical health in local budget decisions in England. Ring-fencing funding, raising awareness among commissioners and improving the evidence base are steps that could contribute to more equitable funding in this area of need.

Author contributions

SR contributed to the study, carried out the data acquisition, conducted the analysis and drafted the manuscript. AT also contributed to the study design, analysis plan, and helped refine the manuscript. MF provided input into the study design and provided feedback on the manuscript. All authors have read and approved the final version.

Funding

This research was funded by the National Institute for Health Research (NIHR) Collaboration for Leadership in Applied Health Research and Care Oxford at Oxford Health NHS Foundation Trust. The views expressed are those of the author(s) and not necessarily those of the NHS, the NIHR or the Department of Health and Social Care.

Competing interests

None declared.

Data sharing statement

All data used in this study is publicly available and can be re-used. See the supplementary file for a full list of data sources.

For peer review only - http://bmjopen.bmj.com/site/about/guidelines.xhtml

Transparency

The lead author (SR) affirms that the manuscript is an honest, accurate, and transparent account of the study being reported; that no important aspects of the study have been omitted; and that any discrepancies are disclosed.

For peer review only

For peer review only - http://bmjopen.bmj.com/site/about/guidelines.xhtml

References

1. Doran CM, Kinchin I. A review of the economic impact of mental illness. *Australian health review : a publication of the Australian Hospital Association* 2017 doi: 10.1071/ah16115 [published Online First: 2017/11/14]
2. OECD. Mental Health and Work: United Kingdom2014.
3. Kessler RC, Amminger GP, Aguilar-Gaxiola S, et al. Age of onset of mental disorders: A review of recent literature. *Current opinion in psychiatry* 2007;20(4):359-64. doi: 10.1097/YCO.0b013e32816ebc8c
4. Patton GC, Sawyer SM, Santelli JS, et al. Our future: a Lancet commission on adolescent health and wellbeing. *The Lancet* 2016;387(10036):2423-78. doi: https://doi.org/10.1016/S0140-6736(16)00579-1
5. Neufeld SAS, Dunn VJ, Jones PB, et al. Reduction in adolescent depression after contact with mental health services: a longitudinal cohort study in the UK. *The Lancet Psychiatry* 2017;4(2):120-27. doi: 10.1016/s2215-0366(17)30002-0
6. Romeo R, Byford S, Knapp M. Annotation: Economic evaluations of child and adolescent mental health interventions: a systematic review. *J Child Psychol Psychiatry* 2005;46(9):919-30. doi: 10.1111/j.1469-7610.2005.00407.x
7. NHS England. Fair Shares: A guide to NHS Allocations, 2017.
8. Earle DJ. Children and young people's mental health British Medical Association, 2016.
9. Hawton K, Saunders KEA, O'Connor RC. Self-harm and suicide in adolescents. *The Lancet* 2012;379(9834):2373-82. doi: 10.1016/s0140-6736(12)60322-5
10. Frith E. Progress and challenges in the transformation of children and young people's mental health care: a report of the Education Policy Institute's Mental Health Commission: Education Policy Institute, 2016.
11. Rutherford CA, Taylor-Robinson D. Wide variation in child mental health spend in England. *BMJ* 2017;356 doi: 10.1136/bmj.j451
12. NHS England. The Five Year Forward View For Mental Health, 2016.
13. NHS England. Children and Young People's Mental Health Services Baselining Report: Local Transformation Plans Review 2015, 2016.
14. Royal College of Physicians RCoP. Smoking and mental health: A joint report by the Royal College of Physicians and the Royal College of Psychiatrists. London: Royal College of Psychiatrists Council Report, 2013.
15. Department for Education. Policy paper: Review of Children in Need Gov.uk2018 [Available from: https://www.gov.uk/government/publications/review-of-children-in-need/review-of-children-in-need accessed 12/11/2018 2018.
16. Children's Commissioner. Lightning Review: Access to Child and Adoloscent Mental Health Services. 2016
17. Royal College of Psychiatrists. The scandal of underfunded child and adolescent mental health services laid bare in new research from the Royal College of Psychiatrists www.rcpsych.ac.uk2016 [accessed May 2018.
18. Hargreaves DS, Lemer C, Ewing C, et al. Measuring and improving the quality of NHS care for children and young people. *Archives of Disease in Childhood* 2018 doi: 10.1136/archdischild-2017-314564
19. Cookson R, McCabe C, Tsuchiya A. Public healthcare resource allocation and the Rule of Rescue. *Journal of medical ethics* 2008;34(7):540-4. doi: 10.1136/jme.2007.021790 [published Online First: 2008/07/02]
20. Zweifel P. The triple challenge of mental health. *Eur J Health Econ* 2018;19(3):309-13. doi: 10.1007/s10198-017-0937-z
21. Memon A, Taylor K, Mohebati LM, et al. Perceived barriers to accessing mental health services among black and minority ethnic (BME) communities: a qualitative study in Southeast England. *BMJ Open* 2016;6(11) doi: 10.1136/bmjopen-2016-012337

For peer review only - http://bmjopen.bmj.com/site/about/guidelines.xhtml

22. Bhatnagar P, Wickramasinghe K, Williams J, et al. The epidemiology of cardiovascular disease in the UK 2014. *Heart* 2015 doi: 10.1136/heartjnl-2015-307516
23. Hu G, Jousilahti P, Barengo NC, et al. Physical Activity, Cardiovascular Risk Factors, and Mortality Among Finnish Adults With Diabetes. *Diabetes Care* 2005;28(4):799-805. doi: 10.2337/diacare.28.4.799
24. Sadler K, Vizard T, Ford T, et al. Mental Health of Children and Young People in England, 2017. 2018
25. Children’s Commissioner. Briefing: Children’s Mental Healthcare in England, 2017.
26. Currie G, Croft C, Chen Y, et al. The capacity of health service commissioners to use evidence: a case study. In: Research HSaD, ed. Southampton (UK): NIHR Journals Library 2018.
27. Klein R. Dimensions of rationing: who should do what? *BMJ : British Medical Journal* 1993;307(6899):309-11.
28. Jan S, Dommers E, Mooney G. A politico-economic analysis of decision making in funding health service organisations. *Social Science & Medicine* 2003;57(3):427-35. doi: https://doi.org/10.1016/S0277-9536(02)00368-4
29. Eddama O, Coast J. Use of economic evaluation in local health care decision-making in England: a qualitative investigation. *Health policy (Amsterdam, Netherlands)* 2009;89(3):261-70. doi: 10.1016/j.healthpol.2008.06.004 [published Online First: 2008/07/29]
30. Sheiner L. Why the Geographic Variation in Health Care Spending Cannot Tell Us Much About the Efficiency or Quality of Our Health Care System. *Brookings Papers on Economic Activity* 2014;45(2 (Fall)):1-72.

For peer review only

For peer review only - http://bmjopen.bmj.com/site/about/guidelines.xhtml

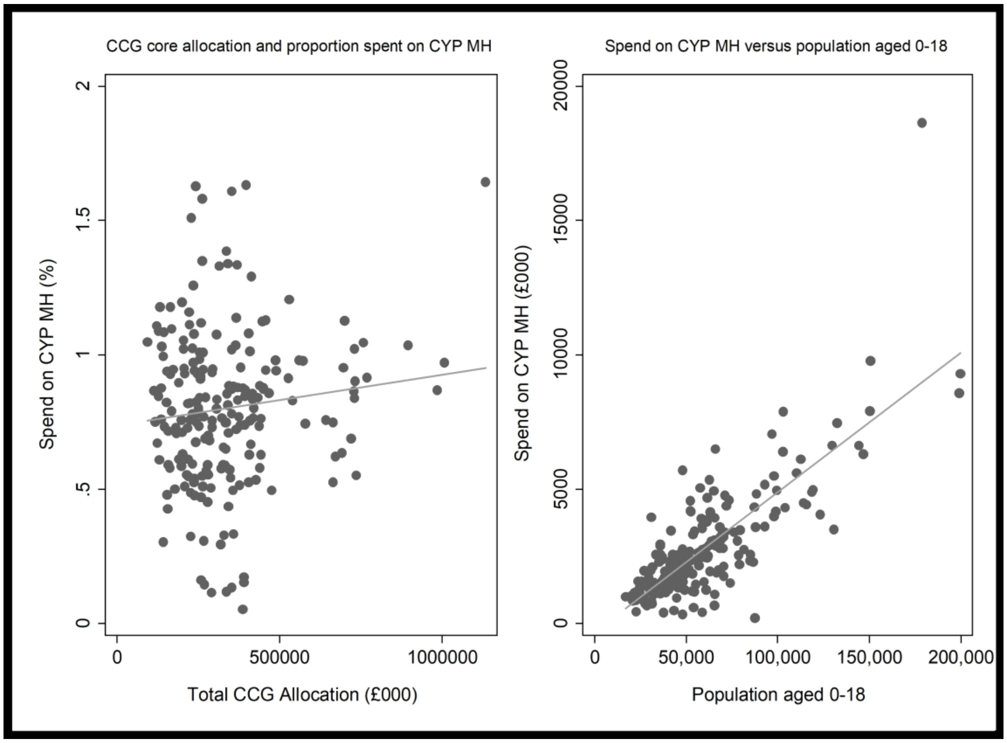

Figure 1 – Scatter plots: Spend on child and young person mental health services

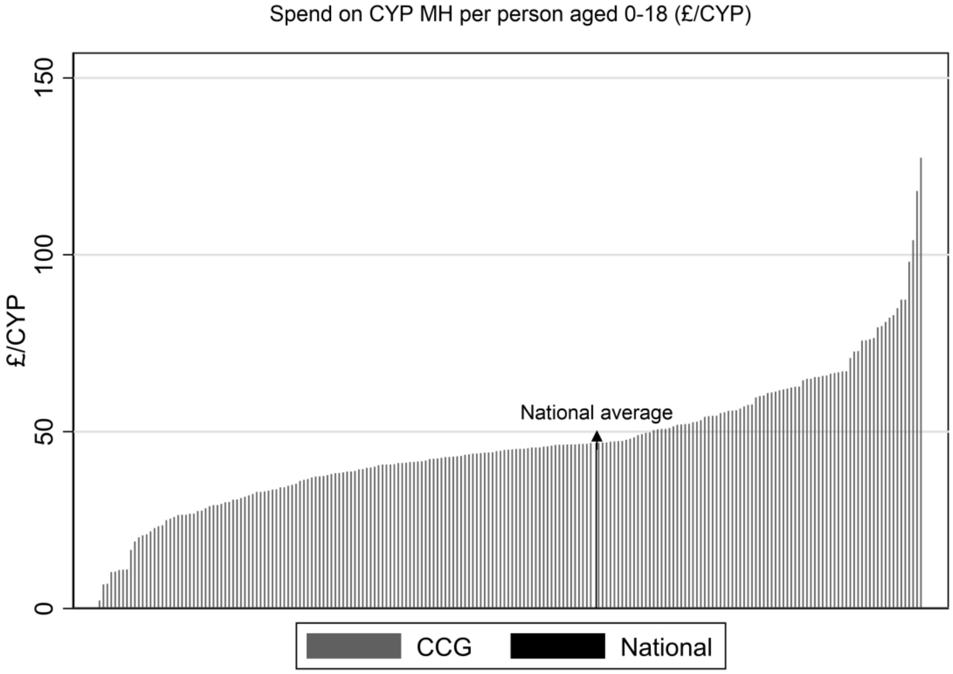

Figure 2 – Spend on child and young person mental health services, by CCG

Appendix

Table 1 - Overview of data

Variable	Category	Year	Source	Note
Spend on CYP MH	Dependent variable	2016/17	Mental Health Five Year Forward View Dashboard	
Estimated prevalence (aged 5-16) of:<br>• any mental health condition<br>• conduct disorder<br>• emotional disorders<br>• hyperkinetic disorders	CYP MH need	2015 (2004 prevalence estimate applied to 2015 population)	PHE Fingertips	
Children in need	CYP MH need	2016/17	Gov.uk, Characteristics of children in need: 2016 to 2017	Estimated from local authority data*
Hospital admissions as a result of self-harm among those aged 0-24	CYP MH need	2016/17	PHE Fingertips	
Hospital admissions for mental health under 18 (rate per population aged 0-18)	CYP MH need	2015/16, 2016/17	PHE Fingertips	
Child Development at age 5	CYP MH need	2013/14	Local Health	
GCSE Achievement 5 A-C	CYP MH need	2013/14	Local Health	
Obese Children (Reception Year)	CYP MH need	2013/14-2015/16	Local Health	
Children with excess weight (Reception Year)	CYP MH need	2013/14-2015/17	Local Health	
Children with excess weight (Year 6)	CYP MH need	2013/14-2015/18	Local Health	
Obese Children Year 6	CYP MH need	2013/14-2015/16	Local Health	
Regular smoker (modelled prevalence)	CYP MH need	2009-2012	Local Health	
Looked after children where there is a cause for concern	CYP MH need	2016/17	PHE Fingertips	Estimated from local authority data*
Children leaving care	CYP MH need	2016/17	PHE Fingertips	Estimated from local authority data*
Children in care	CYP MH need	2016/17	PHE Fingertips	Estimated from local

For peer review only

				authority data*
Children entering youth justice system	CYP MH need	2016	PHE Fingertips	Estimated from local authority data*
Children under 16 in poverty (%)	CYP MH need	2015	PHE Fingertips	Estimated from local authority data*
Family homelessness (%)	CYP MH need	2016/17	PHE Fingertips	Estimated from local authority data*
Not in education, employment or training (%)	CYP MH need	2015	PHE Fingertips	Estimated from local authority data*
Special educational needs (SEN) (rate per population aged 0-18)	CYP MH need	2016	Gov.uk, Special educational needs (SEN)	Estimated from local authority data*
Adult mental health spend (excluding CYP MH, but including learning disabilities)	Adult MH need	2016/17	Mental Health Five Year Forward View Dashboard	
Quality and outcomes framework (QOF) prevalence estimates (%): • Depression (ages 18+) • Severe Mental Illness	Adult MH need	2015/16	NHS Digital	
Mental health total primary care expenditure (£000)	Adult MH need	2015/16	NHS RightCare 'Where to look' packs	
Quality and outcomes framework (QOF) prevalence estimates (%): • Asthma • Cancer • Chronic Kidney Disease (CKD) (ages 18+) • Chronic Obstructive Pulmonary Disease (COPD) • Coronary heart disease (CHD) • Diabetes mellitus (ages 17+) • Dementia • Epilepsy • Learning Disabilities • Heart Failure • Hypertension • Obesity (ages 16+) • Osteoporosis • Palliative care • Peripheral arterial disease • Rheumatoid Arthritis • Stroke and transient ischaemic attack (STIA) • Atrial fibrillation	Physical health need	2015/16	NHS Digital	

For peer review only

1) Spend on physical health conditions (2015/16) – age and sex standardised:<br>○ Asthma (non-elective)<br>○ Cancer:<br>i. Bowel cancer (electives, non-elective)<br>ii. Lung cancer (electives, non-elective)<br>iii. Breast cancer (elective, primary care)<br>○ Endocrine (electives, non-elective, primary care)<br>○ Gastrointestinal (electives, non-elective, primary care)<br>○ Genitourinary (electives, non-elective, primary care)<br>○ Maternity (primary care)<br>○ Musculoskeletal (electives, non-elective, primary care)<br>○ Neurological (electives, non-elective, primary care)<br>○ Respiratory (electives, non-elective, primary care)<br>○ Trauma and injuries (electives, non-elective, primary care)	Physical health need	2015/16	NHS RightCare 'Where to look' packs	
Elective hospital admissions for all causes	Physical health need	2011/12-2015/16	Local Health	
Emergency hospital admissions for all causes	Physical health need	2011/12-2015/16	Local Health	
Indices of multiple deprivation (IMD)	Confounder	2015	Local Health	
Total CCG core budget allocation	Confounder	2016/17, 2017/18	NHS England, Allocations	
BME	Confounder	2011	Local Health	
Population whose ethnicity is not White British	Confounder	2011	Local Health	
Long Term Unemployment	Confounder	2016/17	Local Health	
Unemployment	Confounder	2016/17	Local Health	

*** Mapping Local Authorities to CCGs**

Local Government Association:
https://www.local.gov.uk/sites/default/files/documents/mapping-ccgs-hwbs-and-hwb-507.xls

For peer review only

Table 2 - Descriptive statistics of explanatory factors included in the main regression model

	count	mean	sd	min	max
CYP MH spend per person aged 0-18	209	46.39	18.51	2.25	127.42
Children entering youth justice per 1,000 aged 0-18	204	3.23	1.20	0.98	7.40
Children in need, Primary need: family stress, rate per 100 aged 0-18	204	1.02	0.58	0.11	2.70
Obese Children (Reception Year)	204	9.25	1.58	5.30	13.80
Children with excess weight (Reception Year)	204	22.10	2.35	14.60	27.20
Children in care per 1,000 aged 0-18	204	6.13	2.23	2.15	16.38
Spend on MH per person over 18 (excl. CYP, £10)	209	22.45	5.34	12.66	44.41
All physical health spend per person (£100)	209	5.80	1.01	3.44	9.26
Black and minority ethnic (%)	204	14.03	15.53	1.20	72.20
Long term unemployment (%)	204	3.57	2.54	0.50	14.40

For peer review only

Variation in spend on young mental health across Clinical Commissioning Groups in England: a cross-sectional observational study

Journal:	*BMJ Open*
Manuscript ID	bmjopen-2019-030011.R1
Article Type:	Original research
Date Submitted by the Author:	13-Jun-2019
Complete List of Authors:	Rocks, Stephen; University of Oxford, Health Economics Research Centre<br>Fazel, Mina; University of Oxford, Warneford Hospital,, Psychiatry; University of Oxford<br>Tsiachristas, Apostolos; University of Oxford, Health Economics Research Centre
**Primary Subject Heading**:	Mental health
Secondary Subject Heading:	Health economics, Health policy, Health services research, Mental health
Keywords:	Health economics < HEALTH SERVICES ADMINISTRATION & MANAGEMENT, Organisation of health services < HEALTH SERVICES ADMINISTRATION & MANAGEMENT, MENTAL HEALTH, Child & adolescent psychiatry < PSYCHIATRY

Variation in spend on young mental health across Clinical Commissioning Groups in England: a cross-sectional observational study

Authors

Stephen Rocks[1 x], Mina Fazel[2], Apostolos Tsiachristas[1]

[x] Corresponding author
stephen.rocks@ndph.ox.ac.uk
+44 (0)1865 289470

[1] Health Economics Research Centre, Nuffield Department of Population Health, University of Oxford, Richard Doll Building, Old Road Campus, Oxford OX3 7LF
[2] Department of Psychiatry, University of Oxford, Warneford Hospital, Warneford Ln, Oxford OX3 7JX

Word count – 3,596 (excluding title page, abstract, references, figures, tables, acknowledgements, contributions and references)

ABSTRACT

Objectives

To investigate whether the rate of spend on child and adolescent mental health is influenced by demand for other competing services in local commissioning decisions.

Design

Analysis of spend data by Clinical Commissioning Groups (CCG), including other publicly available data to control for variation in need.

Setting

Local commissioning decisions in the NHS.

Participants

Commissioning of health services across 209 CCGs.

Main outcome measures

Association between the rate of child and adolescent mental health spend and demand for CAMHS, adult mental health services and physical health services after adjusting for confounding factors.

Results

An additional percentage point in the proportion of children in care is associated with 4% higher CYP MH spend per person aged 0-18 (ratio of means: 1.04; 95%CI: 1.00, 1.07). Spending £100 more on

For peer review only - http://bmjopen.bmj.com/site/about/guidelines.xhtml

physical health services was associated with 9% lower spend in CYP MH per person aged 0-18 (ratio of means: 0.91; 95%CI: 0.84, 0.99).

Conclusions

Healthcare commissioners in England face a challenge in balancing competing needs. This paper contributes to our understanding of this by quantifying the possible extent of the trade-off between physical health and CYP MH when allocating budgets. Any attempt to explain the variation in CAMHS spend must also take account of demand for other services.

Strengths and limitations of this study

- This is the first study looking broadly at the factors associated with the commissioning of child and adolescent mental health services.
- A major strength is the incorporation of different area level data.
- There is limited data available for spend at a commissioning level.
- There are concerns over the consistency of published figures for spend on child and young person mental health.
- The analytic approach and incorporation of sensitivity analyses strengthen this study.

review only

For peer review only - http://bmjopen.bmj.com/site/about/guidelines.xhtml

Introduction

Mental health problems impose a significant burden on individuals, their families and society.[1] It is estimated that the costs associated with mental illness in the UK total 4.5 percent of gross domestic product (GDP), driven mainly by productivity loss and opportunity cost of informal care.[2] Childhood and adolescence is crucial to any attempt to alleviate this burden because the majority of lifelong mental illness has started by the age of 17,[3] exerting short and long-term costs that impact significantly across the life course.[4] However, although effective interventions exist,[5] most people do not access mental health interventions during their childhood and adolescence.[3 6] It is estimated that less than a quarter of young people with a mental health problem receive any help from specialist services.[3] This treatment gap reflects a disparity between the needs and resources committed to the mental health of young people.

In England, the decision on how to fund most child and adolescent mental health services (CAMHS) is taken by NHS Clinical Commissioning Groups (CCGs). The level of spend is not predetermined. CCGs are assigned an overall budget based on the physical and mental health care needs of the local population and commission services to meet those needs, but as NHS England states, 'It is for CCGs to decide their priorities for spending'.[7] Analysis by the Royal College of Psychiatrists discovered a stark variation in spend on CAMHS contributing to a "postcode lottery" in access to services.[8]

Variation in spend on CAMHS could be justified if it reflected real differences in need for mental health services among young people, but research suggests it is only weakly associated with deprivation – a proxy for need - with 'stark unexplained variation'.[8] Indeed, recent evidence from England suggests the budget allocation to CAMHS does not reflect levels of psychopathology. For instance, between 2012/13 and 2014/15 there was a sharp rise in referrals to CAMHS alongside a growing number of young people presenting at A&E with self-harm, [9 10] but some local CAMHS budgets were reduced in real terms over the same period.

Instead, it could be that spend on CAMHS reflects spending decisions for other services, either adult mental health or general and acute health services. The aim of this study was to assess the extent of the trade-off that exists between spend on CAMHS and other services. In order to quantify this we analysed the spending decisions of CCGs in England. Amid concerns that CAMHS are under-resourced and that recently earmarked additional national funding for CAMHS may be diverted to other areas,[11] it is important to better understand the funding allocation decisions taken by commissioners.

Methods

Data sources

We accessed publicly available data from the sources described below. Data on spend for child and young person mental health (CYP MH) and adult mental health by CCG (N=209) was available from the Five Year Forward View Mental Health Dashboard (FYFVMHD).[12] Although NHS England reports on 'child and young person' mental health (CYP MH) spend, CCGs spend variable amounts on other services for CYP MH such as acute paediatric care. In this paper, however, we take overall spend as a proxy for local child and adolescent mental health services (CAMHS). Annual spend figures were available for the financial years 2016/17 and 2017/18. CCG population data was sourced from the Office of National Statistics (ONS).

For peer review only - http://bmjopen.bmj.com/site/about/guidelines.xhtml

Other data on CCG areas were identified from a range of publicly available UK data sets, including Public Health Fingertips, the NHS Outcomes Tool, NHS RightCare packs, and Local Health. As far as possible data was used for the same year as CYP MH spend (2016/17). Where this was not available, the nearest possible date was used; 2016 was chosen where data were available for calendar years. When data were reported for local authorities rather than CCGs, approximate values for CCGs were calculated using a 'lookup' file from the Local Government Association, which provides a link between local authority and CCG data. A full list of accessed data with time periods and sources is provided in the supplementary file (Table S1).

Spend on child and young person mental health

CCG reporting on CYP MH spend was introduced as part of the FYFVMHD. This figure covers the majority of CAMHS spend on tiers 2 (early help and targeted services) and 3 (specialised CAMHS services).[13] Spend on specialist eating disorder services is reported separately. CYP MH spend does not include inpatient services, which are commissioned directly by NHS England and therefore not the subject of local allocation decisions, nor other services directly commissioned by other sources such as some children's psychological medicine or liaison services commissioned by acute healthcare trusts.

We used 2016/17 spend in our analysis given the availability of data in other categories of our analysis. Spend was analysed in absolute terms and as a rate per young person aged 0 to 18, using CCG Mid-Year Population Estimates from the Office of National Statistics.

Potential explanatory factors of CAMHS spend variation

We selected a comprehensive set of indicators using a mix of evidence and consultation with health economic and mental health experts.

Category A: Demand for CAMHS

We included 2015 local prevalence estimates for mental health conditions as an indicator of demand for CAMHS. In-patient admissions to psychiatric units among those aged under-18 and rates of self-harm were also included as proxies of need. Other potential explanatory factors included the rate of children "in need",[14] the rate of children in care, as well as a range of broader measures of vulnerability among young people as detailed in Table 1.

Category B: Demand for adult mental health services

For adult mental health, we included 2016/17 mental health spend (excluding spend on children and young people and including primary care spend on mental health) from the FYFVMHD, 2015/16 mental health primary care expenditure from NHS RightCare 'Where to Look' packs and, finally, the Quality and Outcome Frameworks (QOF) prevalence estimates for Depression and Severe Mental Illness from 2015/16.

Category C: Demand for physical health services

The need for physical health spend was proxied by a combination of spend, hospital admissions and prevalence rates. We included a range of physical health spend figures from 2015/16 as reported in NHS RightCare 'Where to Look' packs. In the packs, spend was reported based on major International Classification of Disease (ICD-10) codes, further broken down into elective, non-elective and primary care spend and expressed as standardised rates. To calculate the crude rate of total physical health spend, we summed the numerators (absolute spend) across all physical health

For peer review only - http://bmjopen.bmj.com/site/about/guidelines.xhtml

categories and expressed this as a rate using the respective CCG population. For the more detailed model we summed the numerators for elective, non-elective and primary care spend for each condition and expressed this as a rate using the CCG population. We also included rates of elective and emergency hospital admissions. In addition, QOF prevalence estimates from 2015/16 were used as alternative indicators of demand for physical health care. We chose prevalence estimates of this year because they were available at the point of the commissioning decisions in 2016/17 and were expected to be associated with the variable of interest.

Category D: General factors

More general factors associated with health needs and spend on CYP MH were also included in the analysis. This included the level of deprivation, as measured by the index of multiple deprivation (IMD), unemployment, and long-term unemployment. Research also suggests ethnic minority status may be linked to unmet need - the Children's Commissioner found children of Asian background were under-represented in CAMHS -[15] therefore the proportion of the population from black and minority ethnic (BME) groups was considered. The core allocation per person for each CCG was included to control for differences in budget. We included the difference between what NHS England's allocation formula suggests an area should get (target allocation) and the actual amount allocated in a given year – the 'distance from target allocation'. The two values diverge because of rules limiting the pace of change i.e. the amount by which allocations can change year-on-year. We also included the Market Forces Factor, an index that adjusts for variation in costs, as well as the percent of the population aged 65 years or more and 85 years or more. Finally, we included the rates of overcrowding of households and hospital stays for alcohol related harm.

Table 1 – Summary of potential explanatory factors of CAMHS spend variation

**Category A: Demand for CAMHS**
• Prevalence estimates (% aged 5-16), of: any mental health condition, conduct disorder, emotional disorders, hyperkinetic disorders<br>• Child development at age 5 (%)<br>• GCSE Achievement (%) – attaining 5 A-Cs including English and Maths<br>• Child obesity (%), at: reception year, year 6<br>• Children overweight (%), at: reception year, year 6<br>• Children in need (rate per population aged 0-18): Episodes per year, Children per year, Rate where mental health is a factor, Rate where domestic violence is a factor, Rate where cause is abuse or neglect, Rate where cause is family stress<br>• Special educational needs (SEN) (rate per population aged 0-18)<br>• Hospital admissions for mental health under 18 (rate per person aged 0-18)<br>• Hospital admissions as a result of self-harm, population aged 10-24 (directly standardised rate)<br>• Children in care (rate per population aged 0-18): Children in care, Children leaving care<br>• Looked-after-children where there is a cause for concern (rate per person aged 0-18)<br>• Children under 16 in poverty (%)<br>• Family homelessness (%)<br>• Not in education, employment or training (%)<br>• Rate of first time entrants to the youth justice system
**Category B: Demand for adult mental health services**
• Quality and outcomes framework (QOF) prevalence estimates (%): Depression, Severe Mental Illness

For peer review only - http://bmjopen.bmj.com/site/about/guidelines.xhtml

• Adult mental health spend (excluding CYP MH, but including learning disabilities)<br>• Mental health total primary care expenditure (£000)
**Category C: Demand for physical health services**
• All physical health spend per person (2015/16)<br>• Hospital admissions: Elective and emergency hospital admissions for all causes<br>• Physical health spend per person (2015/16), by condition: Asthma, Cancer (Bowel, Lung, Breast, Other), Circulatory conditions (Cerebrovascular disease, Coronary heart disease, Other), Endocrine (Diabetes, Other), Gastrointestinal (Liver, Lower GI, Upper GI, Other), Genitourinary (Renal, Other), Maternity, Musculoskeletal, Neurological, Osteoarthritis, Osteoporosis, Respiratory (Asthma, Obstructive, Other) Admissions relating to fractures where a fall occurred, Trauma and injuries<br>• Quality and outcomes framework (QOF) prevalence estimates (%) for: Asthma, Cancer, Chronic Kidney Disease (CKD), Chronic Obstructive Pulmonary Disease (COPD), Diabetes, Dementia, Epilepsy, Learning Disabilities, Heart Failure, Hypertension, Obesity, Osteoporosis, Rheumatoid Arthritis, Palliative care, Peripheral arterial disease, Coronary heart disease (CHD), Stroke and transient ischaemic attack (STIA), Atrial fibrillation
**Category D: General factors**
• Index of multiple deprivation (IMD)<br>• Unemployment: Unemployment (%), Long term unemployment (%)<br>• Ethnicity: BME (%), Population whose ethnicity is not 'White UK' (%)<br>• Total CCG core allocation per capita<br>• Distance from target allocation (DfT)<br>• Market Forces Factor<br>• Population aged 65+ (%), Population aged 85+ (%)<br>• Overcrowding<br>• Hospital stays for alcohol related harm

For peer review only

Statistical analysis

To explain variation in spend on CYP MH across CCGs, we reviewed the proportion of CCG budgets spent on CYP MH. We next inspected the association between the proportion spent on CYP MH and the total CCG budget, as well as the association between CYP MH spend per CCG and the population of the CCG aged 0-18.

To investigate statistical associations between CYP MH spend per person aged 0-18 and the explanatory factors identified, we followed a two-step analytical process. First, we selected factors from each category listed in Table 1 that were associated with the dependent variable (i.e. spend on CYP MH per person aged 0-18). To do this, we included all factors in each category in a generalised linear model (GLM) and used likelihood ratio tests to select backwards those factors that gave the best goodness-of-fit of the model to the data – an approach known as "Block-Wise Selection".[16] The order in which the factors were tested by likelihood ratio tests was determined by the p-value from the regression (from largest to smallest p-value).

Second, all selected factors from each category were included in a GLM and the regression coefficients were presented in the exponentiated form to give the ratio of means (i.e. the proportional adjustment in the dependent variable "child and young person mental health spend per person aged 0-18" expected from a 1-unit increase in the covariate). All GLMs were specified with Gamma distribution and log link function because this combination provided the best goodness-of-

For peer review only - http://bmjopen.bmj.com/site/about/guidelines.xhtml

fit based on a Modified Park Test (using the user-command "GLMdiag" in STATA) and the AIC and BIC information criteria. We ran the analysis in the main model featuring the rate of all physical health spend. To understand the relationship in detail, we also ran a second, detailed physical care model, which included spend broken down by category.

Sensitivity analyses

To address the uncertainty in the results, we conducted three sensitivity analyses. First, we removed CCGs with extreme low and high spend on CAMHS from the analysis to address concerns that CAMHS spend figures are incorrectly recorded for some CCGs.[17] The extreme CAMHS spend values (or outliers) were identified using studentized deviance residuals. Second, we added spend on eating disorders in the dependent variable (i.e. spend on CYP MH per person aged 0-18) because some CCGs have been unable to split out these costs due to block contracting and it was unclear if, for some CCGs, these costs were excluded entirely from the dependent variable.[11] Third, QOF prevalence estimates were used as indicators of physical health need instead of spend on physical care because physical health spend data was out of date by a year compared with the CAMHS spend data.

Patient and public involvement

This research was done without patient involvement. Patients were not invited to comment on the study design and were not consulted to develop patient relevant outcomes or interpret the results. Patients were not invited to contribute to the writing or editing of this document for readability or accuracy.

Results

Spend on CYP MH (excluding spend on specialist eating disorder services) accounted on average for 0.8% of CCGs' budget in 2016/17. As shown in Figure 1, it appears that the proportion of CCG budget spent on CYP MH has a weak positive association with the size of the CCG budget and a strong positive association with the size of the CYP population.

Figure 1 – Scatter plots: Spend on child and young person mental health services

The average spend on CYP MH per person aged 0-18 in 2016/17 across 209 CCGs was £46 (SD=£18, min=£2, max=£127) with considerable variation across CCGs in England (Figure 2).

Figure 2 – Spend on child and young person mental health services, by CCG

The results from the main regression analysis are presented in Table 2 and show that an additional percentage point in the proportion of children in care is associated with 4% higher CYP MH spend per person aged 0-18 (ratio of means: 1.04; 95%CI: 1.00, 1.07). By contrast, spending £100 more on physical health services was associated with 9% lower spend in CYP MH per person aged 0-18 (ratio of means: 0.91; 95%CI: 0.84, 0.99). The proportion of the population of black and minority ethnicity (BME) also appeared to be negatively associated with the dependent variable but the magnitude was small (ratio of means: 0.99; 95%CI: 0.98, 1.00). Overall, the main regression model explained 29% of the variation in CYP MH spend per person. The summary statistics for the explanatory factors included in the main regression model are presented in the supplementary file (table 2).

For peer review only - http://bmjopen.bmj.com/site/about/guidelines.xhtml

Regarding the first sensitivity analysis, 7 CCGs were excluded because they spent less than £11 per person aged 0-18 on CYP MH and 2 CCGs were excluded because they spent above £117 per head. The results of this sensitivity analysis as well as the sensitivity analysis where spend on eating disorders was added in the dependent variable were very similar to the results of the main analysis. The most noticeable difference was that spending £10 more on adult mental health was significantly and positively associated with 2% (ratio of means: 1.02; 95%CI: 1.00, 1.03) and 1% (ratio of means: 1.01; 95%CI: 1.00, 1.03) more spend on CYP MH spend per person aged 0-18, respectively. Also, the negative association between spend on physical health with the dependent variable was reduced from 9% in the main analysis to 7% (ratio of means: 0.93; 95%CI: 0.87, 1.00) and 8% (ratio of means: 0.92; 95%CI: 0.86, 0.98), respectively.

For peer review only

For peer review only - http://bmjopen.bmj.com/site/about/guidelines.xhtml

Table 2 – Association of CYP MH spend per person aged 0-18 with explanatory factors (main model)

	**Main analysis** Ratio of Means (SE) [95% CI]	**SA 1 – excluding outliers** Ratio of Means (SE) [95% CI]	**SA 2 – including eating disorders** Ratio of Means (SE) [95% CI]
Children entering youth justice per 1000 aged 0-18	1.04 (0.03) [0.99,1.09]	1.02 (0.02) [0.97,1.07]	1.03 (0.03) [0.98,1.09]
Children in need (family stress), rate per 100 aged 0-18	1.05 (0.05) [0.96,1.15]	1.04 (0.04) [0.97,1.13]	1.06 (0.05) [0.98,1.16]
Obese Children (Reception Year)	0.94 (0.05) [0.84,1.05]	0.95 (0.05) [0.86,1.05]	0.94 (0.05) [0.84,1.05]
Children with excess weight (Reception Year)	1.05 (0.04) [0.98,1.12]	1.05 (0.03) [0.99,1.11]	1.04 (0.03) [0.98,1.11]
Children in care per 1,000 aged 0-18	1.04* (0.02) [1.00,1.07]	1.03* (0.01) [1.00,1.05]	1.04* (0.02) [1.01,1.07]
Spend on MH per person over 18 (excl. CYP, £10)	1.01 (0.01) [1.00,1.03]	1.02** (0.01) [1.00,1.03]	1.01* (0.01) [1.00,1.03]
All physical health spend per person (£100)	0.91** (0.04) [0.84,0.99]	0.93* (0.03) [0.87,1.00]	0.92** (0.04) [0.85,1.00]
% Black and minority ethnic	0.99** (0.00) [0.98,1.00]	0.99* (0.00) [0.98,1.00]	0.99* (0.00) [0.99,1.00]
Overcrowding	1.01 (0.01) [0.99, 1.03]	1.01 (0.01) [0.99, 1.02]	1.01 (0.01) [0.99, 1.02]
Long term unemployment	1.03 (0.02) [1.00,1.06]	1.02 (0.01) [0.99,1.04]	1.02 (0.01) [0.99,1.05]
Observations	199	190	199
AIC	1935	1855	1963
BIC	1971	1890	2000
Pseudo R-squared	.29	.31	.28

CI = Confidence Intervals, SA = Sensitivity analysis; * p<0.05, ** p<0.01, *** p<0.001

For peer review only

For peer review only - http://bmjopen.bmj.com/site/about/guidelines.xhtml

The results from the detailed physical care regression model are presented in Table 3 and show that only spend on circulatory conditions (0.98; 95%CI: 0.96, 0.99) and the proportion BME (0.99; 95%CI: 0.98, 1.00) were associated with the rate of spend on CYP MH. Specifically, an additional pound spent on 'other circulatory conditions' per person was associated with a 2% decrease in spend per head on CYP MH. When including QOF prevalence estimates in the detailed model instead of spend on physical health (third sensitivity analysis), there were no significant associations, albeit the point estimates for cancer, diabetes and COPD were each negative.

Table 3 – Association of CYP MH Spend per person aged 0-18 with explanatory factors (detailed physical care model)

	**Detailed spend** Ratio of Means (SE) [95% CI]	**SA 3 – QOF prevalence** Ratio of Means (SE) [95% CI]
Children entering youth justice per 1000 aged 0-18	1.03 (0.03) [0.97,1.09]	1.04 (0.03) [0.99,1.10]
Children in need (family stress), rate per 100 aged 0-18	1.07 (0.05) [0.98,1.18]	1.07 (0.05) [0.97,1.17]
Obese Children (Reception Year)	0.95 (0.06) [0.84,1.07]	0.96 (0.06) [0.85,1.08]
Children with excess weight (Reception Year)	1.04 (0.04) [0.97,1.12]	1.04 (0.04) [0.98,1.12]
Children in care per 1,000 aged 0-18	1.03 (0.02) [1.00,1.07]	1.02 (0.02) [0.99,1.06]
Spend on MH per person over 18 (excl. CYP, £10)	1.01 (0.01) [1.00,1.02]	1.01 (0.01) [1.00,1.02]
Spend on circulatory disease per person: coronary heart disease	1.01 (0.01) [0.98,1.03]	
Spend on circulatory disease per person: other	0.98** (0.01) [0.97,0.99]	
Spend on respiratory disease per person: obstructive conditions	1.01 (0.02) [0.98,1.04]	
Spend on cancer per person: bowel cancer	1.02 (0.04) [0.95,1.10]	
Spend on maternity per person	1.18 (0.10) [0.99,1.40]	
% Black and minority ethnic	0.99* (0.00) [0.98,1.00]	0.99 (0.01) [0.98,1.01]
Overcrowding	1.01 (0.01) [0.99, 1.03]	1.01 (0.01) [0.99, 1.02]
Long term unemployment	1.02 (0.02) [0.99,1.05]	1.03 (0.02) [1.00,1.06]
Cancer prevalence - 18+, QOF		0.93 (0.09) [0.77,1.13]
Diabetes prevalence - 18+, QOF		0.94 (0.03) [0.86,1.03]
COPD prevalence, QOF		0.98 (0.09) [0.82,1.18]
Observations	199	199
AIC	1942	1939
BIC	1991	1981
Pseudo R-squared	.32	.29

CI = Confidence Intervals, SA = Sensitivity analysis; * p<0.05, ** p<0.01, *** p<0.001

Discussion

Healthcare commissioners in England face a challenge in balancing competing needs. This paper contributes to our understanding of this by quantifying the bi-lateral associations, or trade-offs, with CYP MH spend when allocating budgets. Our main findings are that per capita spend on CYP MH was positively associated with need, the level of children in need stands out as significant among the variables selected; positively associated with spend on adult mental health; but negatively associated with spend on physical health and the proportion of the population which is from black and minority ethnic backgrounds.

In principle, with homogenous preferences and perfect information on both the needs and optimal treatments to inform the allocation of budgets, we would not expect a trade-off with physical health to exist once accounting for variation in need and the size of the population. Variation in need alone would explain differences in spend on CYP MH. In practice, the decentralised system of healthcare commissioning in England offers a number of possible reasons for the identified association, for instance: the configuration of local services may differ; there may be variation in patient preferences; or commissioners might have different preferences when allocating funding.[18] Decisions are also constrained by history: resistance to large-scale disinvestment means that in any

For peer review only - http://bmjopen.bmj.com/site/about/guidelines.xhtml

given year the scope for reallocating budgets across competing claims is likely to be marginal at best.[19]

However, concerns persist that commissioners do not readily prioritise CAMHS. Indeed, CAMHS has been described as the 'Cinderella of the Cinderella service' in that despite repeated promises of additional funding it has arguably remained under-resourced relative to levels of need.[9] Although the associations identified as part of this paper can do nothing to prove or disprove this, claims that national funding earmarked for CAMHS may have been diverted to other services raises the question as to whether incentives or biases exist that give priority to physical health.[11] We explore possible reasons below.

Firstly, spending decisions may be biased towards a 'rule of rescue'. This predicts that spend will gravitate towards cases of immediate and pressing need and away from what might be considered preventative.[20] It may be that CYP MH is perceived as an area where rationing by deterrence or delay is more acceptable than in other areas, especially as some of the benefits are long-term. Indeed, long waits for CAMHS appear to evidence this.[11]

Secondly, the lower quality and visibility of data relating to CYP MH compared to care for adults may have influenced spending decisions.[21] Historically, there has been more systematic and thorough recording and measurement of processes relating to acute health conditions such as A&E waiting times and hospital length of stay. By contrast, commissioners have had a relative paucity of data on process and outcomes for CAMHS, which may influence allocation decisions.

Thirdly, the general level of awareness or stigma around mental health may also contribute to low prioritisation of CYP MH.[22] The positive association between spend on adult and CYP mental health found in this paper could be explained by commissioners with increased awareness or knowledge of mental health spending more on mental health services for both CYP and adults.[23] Furthermore, the negative association of spend on CYP MH with the proportion of the BME population found in this study may reflect lower levels of awareness of mental health in BME communities.[24]

Finally, as has been argued elsewhere, financial incentives exist that encourage the adoption of new technologies in healthcare.[25] New technologies are one of the main drivers of spending in the NHS.[26] The lobbying for and adoption of new technologies may favour interventions like innovations in surgical instruments and certain drugs (e.g. those used in oncology) over interventions which are primarily labour-intensive, such as those in CAMHS.

We have been concerned with trade-offs, but it would be misleading to posit physical and mental health purely as substitutes when there are complementarities and services may benefit from being more closely integrated. Good mental health can support physical health and vice versa.[27] Also, more efficient services anywhere in the system could help free resources. For instance, in our detailed spend model we found that spend on CYP MH was negatively associated with spend on some circulatory conditions, one of the highest spend disease groups in the UK.[28] Better prevention and early intervention in the community for these diseases may release resources to help meet the increasing demand for mental health services and in particular CAMHS.

This research has highlighted the extent of the trade-off between physical health spend and spend on CYP MH. In light of concerns that CAMHS is not given the priority it merits, we have also

For peer review only - http://bmjopen.bmj.com/site/about/guidelines.xhtml

For peer review only

suggested some factors that could bias spend towards physical health. We would recommend addressing the inequality in the availability and quality of data for CYP MH to help minimise, or at least make more explicit, unwarranted variation in spend on CAMHS. Steps are already being taken: recently published estimates of the prevalence of mental health disorders among young people may better capture variation in need;[29] CYP MH spend is being monitored as part of the FYFVMH dashboard; and new targets are being introduced, such as for access rates and waiting times, to help commissioners understand how well services are performing.[12] Future research should focus on the outcomes arising from different levels of investment in CAMHS and other services that support young people.

Strengths and limitations

The main limitation of this paper is the availability of data. Limited to a single year of spending, this paper can provide evidence only of association and not causality. There was also a lack of contemporaneous and consistent data. In particular, spend on physical health conditions was from 2015/16, albeit the distribution of spend across different programmes is unlikely to have altered dramatically. Other indicators are not available at CCG level but are estimated from local authority data, specifically this includes a range of factors explaining demand for CYP MH services. Finally, as highlighted above, there are concerns over the consistency and accuracy of the figures published for spend on CYP MH, with challenges in breaking out different facets of expenditure. This is addressed by the sensitivity analyses included.

The major strength of this study is how we have been able to use and combine different area level data from a range of datasets, including efforts to incorporate local authority data to understand factors driving need for CYP MH services. Recent research into variation in Medicare spend in the USA found that area level factors were the most appropriate way to analyse such variation.[30] Seeking a parsimonious model amid the array of available data, we applied a stepwise selection procedure. The main criticism of this set of procedures is that they are too data driven. In order to overcome this, we took a block-wise approach.[16] This meant we retained the theoretical foundation outlined, while still being data driven in our attempts to limit the number of independent variables included. The incorporation of sensitivity analyses to address uncertainty in the results also strengthen this study.

Conclusions

Healthcare commissioners in England face a challenge in balancing competing needs. This paper contributes to our understanding of this by quantifying the bilateral associations with CYP MH spend when allocating budgets. Any attempt to explain the variation in CAMHS spend must also take account of demand for other services.

Author contributions

SR carried out the data acquisition, conducted the analysis and drafted the manuscript. AT also contributed to the study design, analysis plan, and helped refine the manuscript. MF provided input into the study design and provided feedback on the manuscript. All authors have read and approved the final version.

For peer review only - http://bmjopen.bmj.com/site/about/guidelines.xhtml

Funding

This research was funded by the National Institute for Health Research (NIHR) Collaboration for Leadership in Applied Health Research and Care Oxford at Oxford Health NHS Foundation Trust. The views expressed are those of the author(s) and not necessarily those of the NHS, the NIHR or the Department of Health and Social Care.

Competing interests

None declared.

Data sharing statement

All data used in this study is publicly available and can be re-used. See the supplementary file for a full list of data sources.

Ethics

This study uses publicly available data only and therefore further ethical approval was not required.

Transparency

The lead author (SR) affirms that the manuscript is an honest, accurate, and transparent account of the study being reported; that no important aspects of the study have been omitted; and that any discrepancies are disclosed.

For peer review only

References

1. Doran CM, Kinchin I. A review of the economic impact of mental illness. *Australian health review : a publication of the Australian Hospital Association* 2017 doi: 10.1071/ah16115 [published Online First: 2017/11/14]
2. OECD. Mental Health and Work: United Kingdom2014.
3. Kessler RC, Amminger GP, Aguilar-Gaxiola S, et al. Age of onset of mental disorders: A review of recent literature. *Current opinion in psychiatry* 2007;20(4):359-64. doi: 10.1097/YCO.0b013e32816ebc8c
4. Patton GC, Sawyer SM, Santelli JS, et al. Our future: a Lancet commission on adolescent health and wellbeing. *The Lancet* 2016;387(10036):2423-78. doi: https://doi.org/10.1016/S0140-6736(16)00579-1
5. Neufeld SAS, Dunn VJ, Jones PB, et al. Reduction in adolescent depression after contact with mental health services: a longitudinal cohort study in the UK. *The Lancet Psychiatry* 2017;4(2):120-27. doi: 10.1016/s2215-0366(17)30002-0
6. Romeo R, Byford S, Knapp M. Annotation: Economic evaluations of child and adolescent mental health interventions: a systematic review. *J Child Psychol Psychiatry* 2005;46(9):919-30. doi: 10.1111/j.1469-7610.2005.00407.x
7. NHS England. Fair Shares: A guide to NHS Allocations, 2017.
8. Rutherford CA, Taylor-Robinson D. Wide variation in child mental health spend in England. *BMJ* 2017;356 doi: 10.1136/bmj.j451
9. Earle DJ. Children and young people's mental health British Medical Association, 2016.
10. Hawton K, Saunders KEA, O'Connor RC. Self-harm and suicide in adolescents. *The Lancet* 2012;379(9834):2373-82. doi: 10.1016/s0140-6736(12)60322-5
11. Frith E. Progress and challenges in the transformation of children and young people's mental health care: a report of the Education Policy Institute's Mental Health Commission: Education Policy Institute, 2016.
12. NHS England. The Five Year Forward View For Mental Health, 2016.
13. NHS England. Children and Young People's Mental Health Services Baselining Report: Local Transformation Plans Review 2015, 2016.
14. Department for Education. Policy paper: Review of Children in Need Gov.uk2018 [Available from: https://www.gov.uk/government/publications/review-of-children-in-need/review-of-children-in-need accessed 12/11/2018 2018.
15. Children's Commissioner. Lightning Review: Access to Child and Adoloscent Mental Health Services. 2016
16. Jeon J. The strengths and limitations of the statistical modeling of complex social phenomenon: Focusing on SEM, path analysis, or multiple regression models. *Int J Soc Behav Educ Econ Bus Ind Eng* 2015;9(5):1594-602.
17. Royal College of Psychiatrists. The scandal of underfunded child and adolescent mental health services laid bare in new research from the Royal College of Psychiatrists www.rcpsych.ac.uk2016 [accessed May 2018.
18. Appleby J, Raleigh V, Frosini F, et al. Variations in health care. *The good, the bad and the inexplicable London: The King's Fund* 2011
19. Klein R. Dimensions of rationing: who should do what? *BMJ : British Medical Journal* 1993;307(6899):309-11.
20. Cookson R, McCabe C, Tsuchiya A. Public healthcare resource allocation and the Rule of Rescue. *Journal of medical ethics* 2008;34(7):540-4. doi: 10.1136/jme.2007.021790 [published Online First: 2008/07/02]
21. Hargreaves DS, Lemer C, Ewing C, et al. Measuring and improving the quality of NHS care for children and young people. *Archives of Disease in Childhood* 2018 doi: 10.1136/archdischild-2017-314564

For peer review only - http://bmjopen.bmj.com/site/about/guidelines.xhtml

22. Zweifel P. The triple challenge of mental health. *Eur J Health Econ* 2018;19(3):309-13. doi: 10.1007/s10198-017-0937-z
23. Foy AJ, Filippone EJ. The case for intervention bias in the practice of medicine. *Yale J Biol Med* 2013;86(2):271-80.
24. Memon A, Taylor K, Mohebati LM, et al. Perceived barriers to accessing mental health services among black and minority ethnic (BME) communities: a qualitative study in Southeast England. *BMJ Open* 2016;6(11) doi: 10.1136/bmjopen-2016-012337
25. Prasad V, Cifu A. Medical reversal: why we must raise the bar before adopting new technologies. *Yale J Biol Med* 2011;84(4):471-78. [published Online First: 2011/12/]
26. Appleby J. Rises in healthcare spending: where will it end? *BMJ : British Medical Journal* 2012;345:e7127. doi: 10.1136/bmj.e7127
27. Ohrnberger J, Fichera E, Sutton M. The relationship between physical and mental health: A mediation analysis. *Social Science & Medicine* 2017;195:42-49. doi: https://doi.org/10.1016/j.socscimed.2017.11.008
28. Bhatnagar P, Wickramasinghe K, Williams J, et al. The epidemiology of cardiovascular disease in the UK 2014. *Heart* 2015 doi: 10.1136/heartjnl-2015-307516
29. Sadler K, Vizard T, Ford T, et al. Mental Health of Children and Young People in England, 2017. 2018
30. Sheiner L. Why the Geographic Variation in Health Care Spending Cannot Tell Us Much About the Efficiency or Quality of Our Health Care System. *Brookings Papers on Economic Activity* 2014;45(2 (Fall)):1-72.

For peer review only

For peer review only - http://bmjopen.bmj.com/site/about/guidelines.xhtml

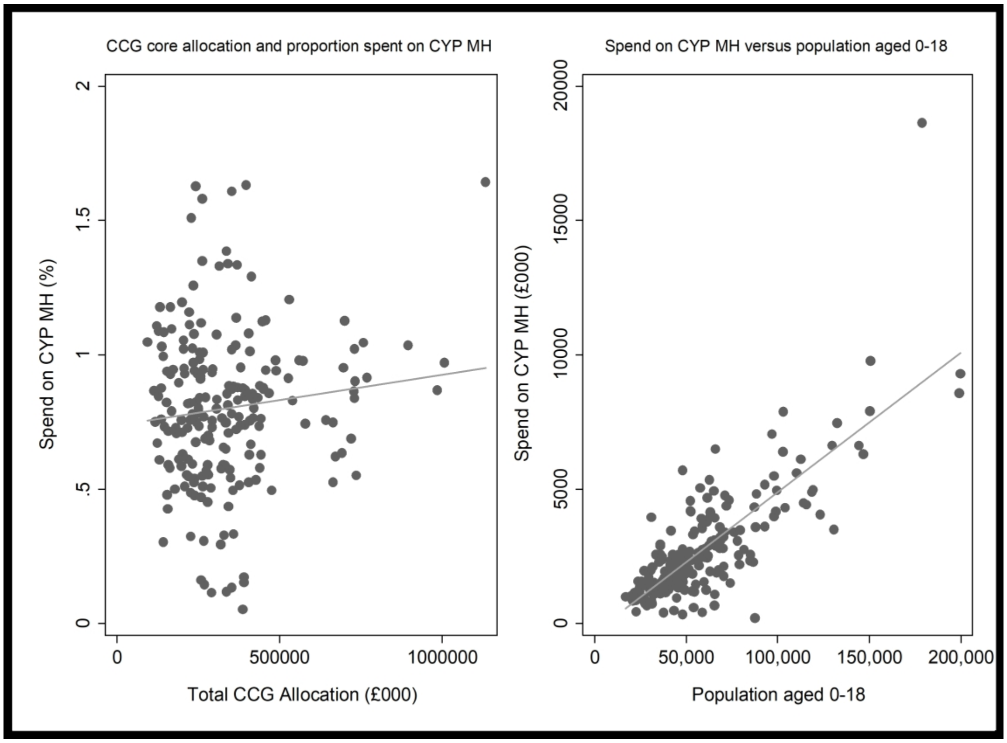

Figure 1 – Scatter plots: Spend on child and young person mental health services

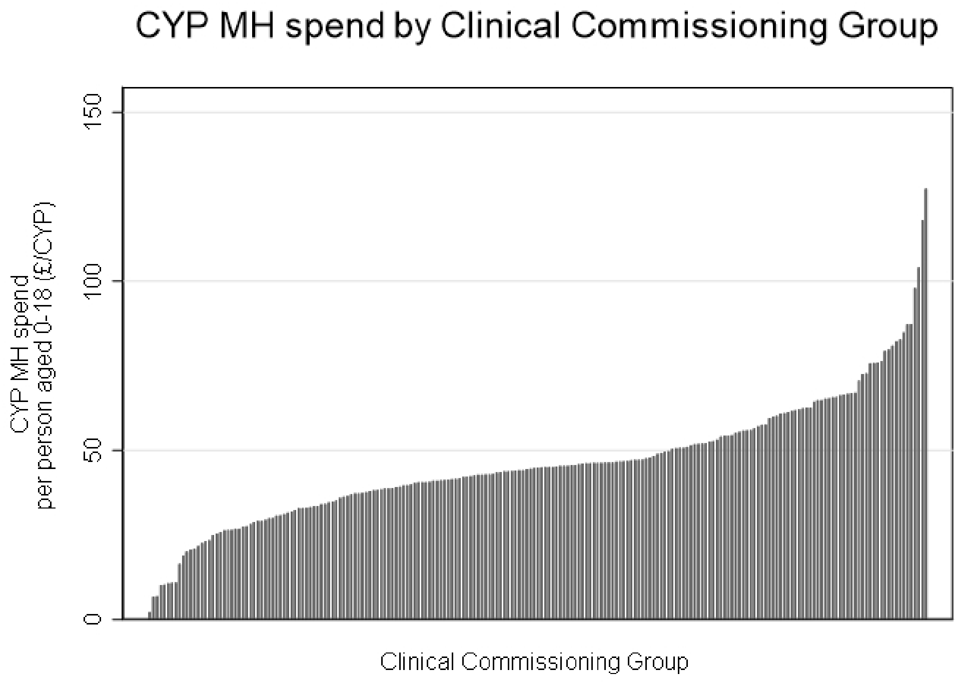

Caption : Figure 2 – Spend on child and young person mental health services, by CCG

240x174mm (72 x 72 DPI)

Appendix

Table 1 - Overview of data

Variable	Category	Year	Source	Note
Spend on CYP MH	Dependent variable	2016/17	Mental Health Five Year Forward View Dashboard	
Estimated prevalence (aged 5-16) of:<br>• any mental health condition<br>• conduct disorder<br>• emotional disorders<br>• hyperkinetic disorders	CYP MH need	2015 (2004 prevalence estimate applied to 2015 population)	PHE Fingertips	
Children in need	CYP MH need	2016/17	Gov.uk, Characteristics of children in need: 2016 to 2017	Estimated from local authority data*
Hospital admissions as a result of self-harm among those aged 0-24	CYP MH need	2016/17	PHE Fingertips	
Hospital admissions for mental health under 18 (rate per population aged 0-18)	CYP MH need	2015/16, 2016/17	PHE Fingertips	
Child Development at age 5	CYP MH need	2013/14	Local Health	
GCSE Achievement 5 A-C	CYP MH need	2013/14	Local Health	
Obese Children (Reception Year)	CYP MH need	2013/14-2015/16	Local Health	
Children with excess weight (Reception Year)	CYP MH need	2013/14-2015/17	Local Health	
Children with excess weight (Year 6)	CYP MH need	2013/14-2015/18	Local Health	
Obese Children Year 6	CYP MH need	2013/14-2015/16	Local Health	
Regular smoker (modelled prevalence)	CYP MH need	2009-2012	Local Health	
Looked after children where there is a cause for concern	CYP MH need	2016/17	PHE Fingertips	Estimated from local authority data*
Children leaving care	CYP MH need	2016/17	PHE Fingertips	Estimated from local authority data*
Children in care	CYP MH need	2016/17	PHE Fingertips	Estimated from local authority data*
Children entering youth justice system	CYP MH need	2016	PHE Fingertips	Estimated from local authority data*
Children under 16 in poverty (%)	CYP MH need	2015	PHE Fingertips	Estimated from local authority data*
Family homelessness (%)	CYP MH need	2016/17	PHE Fingertips	Estimated from local authority data*

For peer review only

Not in education, employment or training (%)	CYP MH need	2015	PHE Fingertips	Estimated from local authority data*
Special educational needs (SEN) (rate per population aged 0-18)	CYP MH need	2016	Gov.uk, Special educational needs (SEN)	Estimated from local authority data*
Adult mental health spend (excluding CYP MH, but including learning disabilities)	Adult MH need	2016/17	Mental Health Five Year Forward View Dashboard	
Quality and outcomes framework (QOF) prevalence estimates (%):<br>• Depression (ages 18+)<br>• Severe Mental Illness	Adult MH need	2015/16	NHS Digital	
Mental health total primary care expenditure (£000)	Adult MH need	2015/16	NHS RightCare 'Where to look' packs	
Quality and outcomes framework (QOF) prevalence estimates (%):<br>• Asthma<br>• Cancer<br>• Chronic Kidney Disease (CKD) (ages 18+)<br>• Chronic Obstructive Pulmonary Disease (COPD)<br>• Coronary heart disease (CHD)<br>• Diabetes mellitus (ages 17+)<br>• Dementia<br>• Epilepsy<br>• Learning Disabilities<br>• Heart Failure<br>• Hypertension<br>• Obesity (ages 16+)<br>• Osteoporosis<br>• Palliative care<br>• Peripheral arterial disease<br>• Rheumatoid Arthritis<br>• Stroke and transient ischaemic attack (STIA)<br>• Atrial fibrillation	Physical health need	2015/16	NHS Digital	
1) Spend on physical health conditions (2015/16) – age and sex standardised:<br>○ Asthma (non-elective)<br>○ Cancer:<br>i. Bowel cancer (electives, non-elective)<br>ii. Lung cancer (electives, non-elective)<br>iii. Breast cancer (elective, primary care)<br>○ Endocrine (electives, non-elective, primary care)<br>○ Gastrointestinal (electives, non-elective, primary care)<br>○ Genitourinary (electives, non-elective, primary care)<br>○ Maternity (primary care)<br>○ Musculoskeletal (electives, non-elective, primary care)<br>○ Neurological (electives, non-elective, primary care)<br>○ Respiratory (electives, non-elective, primary care)<br>○ Trauma and injuries (electives, non-elective, primary care)	Physical health need	2015/16	NHS RightCare 'Where to look' packs	
Elective hospital admissions for all causes	Physical health need	2011/12-2015/16	Local Health	
Emergency hospital admissions for all causes	Physical health need	2011/12-2015/16	Local Health	
Indices of multiple deprivation (IMD)	Confounder	2015	Local Health	

For peer review only

Total CCG core budget allocation	Confounder	2016/17, 2017/18	NHS England, Allocations	
BME	Confounder	2011	Local Health	
Population whose ethnicity is not White British	Confounder	2011	Local Health	
Long Term Unemployment	Confounder	2016/17	Local Health	
Unemployment	Confounder	2016/17	Local Health	
% Population aged 65+	Confounder	2016/17	Local Health	
% Population aged 85+	Confounder	2016/17	Local Health	
Fuel Poverty	Confounder	2016/17	Local Health	
General Health bad or very bad	Confounder	2016/17	Local Health	
Hospital stays for alcohol related harm	Confounder	2016/17	Local Health	
Overcrowding	Confounder	2016/17	Local Health	
MFF Index	Confounder	2016/17	NHS England, Allocations	

*** Mapping Local Authorities to CCGs**

Local Government Association:
https://www.local.gov.uk/sites/default/files/documents/mapping-ccgs-hwbs-and-hwb-507.xls

For peer review only

Table 2 - Descriptive statistics of explanatory factors included in the main regression model

	count	mean	sd	min	max
CYP MH spend per person aged 0-18	209	46.39	18.51	2.25	127.42
Children entering youth justice per 1,000 aged 0-18	204	3.23	1.20	0.98	7.40
Children in need, Primary need: family stress, rate per 100 aged 0-18	204	1.02	0.58	0.11	2.70
Obese Children (Reception Year)	204	9.25	1.58	5.30	13.80
Children with excess weight (Reception Year)	204	22.10	2.35	14.60	27.20
Children in care per 1,000 aged 0-18	204	6.13	2.23	2.15	16.38
Spend on MH per person over 18 (excl. CYP, £10)	209	22.45	5.34	12.66	44.41
All physical health spend per person (£100)	209	5.80	1.01	3.44	9.26
Black and minority ethnic (%)	204	14.03	15.53	1.20	72.20
Overcrowding	204	8.68	7.01	2.70	34.90
Long term unemployment (%)	204	3.57	2.54	0.50	14.40

For peer review only
